# Visual Outcome and Related Factors in Bilateral Total Congenital Cataract Patients: A Prospective Cohort Study

**DOI:** 10.1038/srep31307

**Published:** 2016-08-03

**Authors:** Li Zhang, Xiaohang Wu, Duoru Lin, Erping Long, Zhenzhen Liu, Qianzhong Cao, Jingjing Chen, Xiaoyan Li, Zhuoling Lin, Lixia Luo, Hui Chen, Wu Xiang, Jinchao Liu, Xuhua Tan, Bo Qu, Haotian Lin, Weirong Chen, Yizhi Liu

**Affiliations:** 1 State Key Laboratory of Ophthalmology, Zhongshan Ophthalmic Center, Sun Yat-sen University, Guangzhou, Guangdong, 510060, People’s Republic of China

## Abstract

This study is to evaluate the visual outcome and identify its crucial related factors in children undergoing cataract surgery for bilateral total congenital cataract (CC). This prospective study included consecutive bilateral total cataract patients undergoing primary surgery at Zhongshan Ophthalmic Center (ZOC), Guangzhou, China from Jan 2010 to May 2014. Visual outcome was estimated by best-corrected visual acuity (BCVA) at last follow-up. Potential related factors, including gender, age at last follow-up, age at primary surgery, surgical procedure, postoperative complications (PCs), frequency of follow-up and changes in spectacles were evaluated. Eighty-eight children (176 eyes) were included in the cohort. The mean post-operative BCVA (logMAR) was 1.07 ± 0.53 at the mean follow-up duration 31.07 ± 19.36 months. Multivariable generalized estimating equations (GEEs) showed BCVA was significantly associated with PCs, age at last follow-up and age at primary surgery. Partial correlation analysis indicated age at primary surgery was positively correlated with BCVA controlling for the other factors, both for the whole age range (R = 0.415, P < 0.001) and age >6 months (R = 0.867, P < 0.001). Better visual acuity was related to early primary surgery and low PC occurrence in children with bilateral total CC. Timely surgical intervention and strict control of PCs would be potential steps to achieving better visual outcome.

Congenital cataract (CC) is one of the primary causes of preventable childhood blindness, with an estimated global prevalence ranging from 1 to 15 per 10,000 children^1^,^2^. Although visual acuity can be surgically restored in children with CC, there is great variability in visual outcomes following cataract surgery; some children achieve good postoperative visual acuity, whereas others obtain poor visual outcomes. It has been reported that 5.3% (4/76) of eyes achieve a best-corrected visual acuity (BCVA) of greater than 1.0 and that 7.9% (6/76) of eyes achieve a BCVA of less than 0.1 after bilateral cataract extraction and secondary intraocular lens (IOL) implantation^3^.

Over the past decade, various studies have assessed visual outcome following bilateral CC, but the majority of these studies have been small sample sized series^4^,^5^,^6^. Although it has been reported that numerous factors may affect the final visual outcome after CC surgery^3^,^7^,^8^,^9^,^10^,^11^,^12^,^13^,^14^,^15^,^16^, the crucial factors remain uncertain. A better understanding of the effects of the related factors on visual outcome, considering both direction and amplitude in children with CC, is the fundamental step toward vision restoration.

In the present study, we for the first time conducted a prospective cohort study to follow up and evaluate related factors that might affect the visual outcomes of bilateral CC patients who underwent primary cataract surgery. The results of this study will be of clinical significance to CC management, considering the surgical timing, approach, and control of complications, to achieve an ideal visual outcome.

## Results

### Patient Recruitment and Demographics

**Figure 1:**
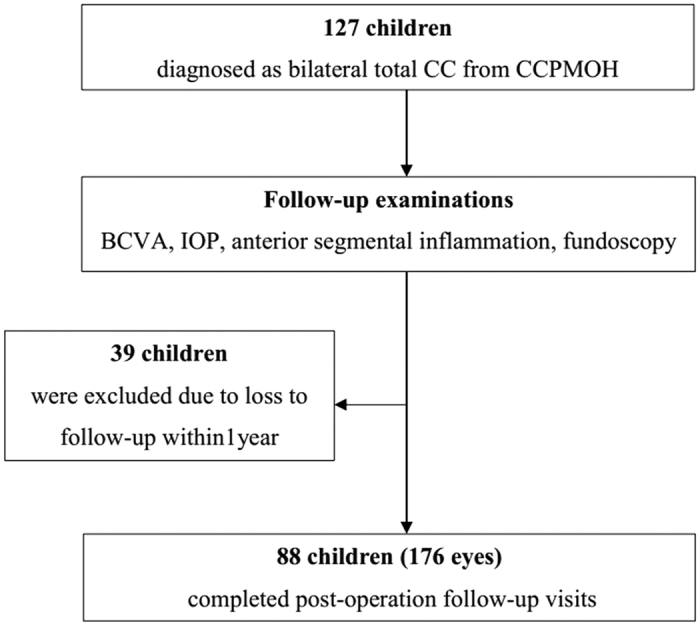
Flowchart of patient selection. CC = Congenital cataract; CCPMOH = Childhood Cataract Program of the Chinese Ministry of Health; BCVA = Best-corrected visual acuity.

**Figure 2:**
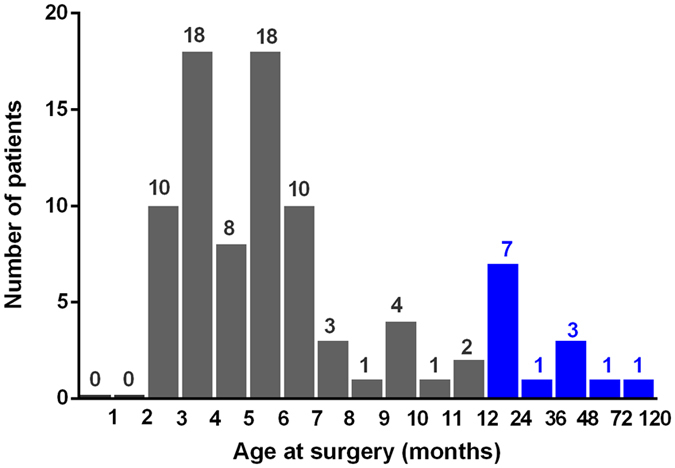
The distribution of included CC patients with different age periods. Nearly 85.2% (75/88) of the included CC patients were younger than 1 year old.

We screened 127 consecutive children from the CCPMOH who presented with bilateral total CC (Fig. 1). After the screening, 39 children (39/127, 30.71%) were excluded because they were unable to complete 1 year of follow-up according to our protocol. Thus, in total, 176 eyes of 88 postoperative children (88/127, 69.29%) completed the follow-up visits. No significant differences were detected between the included patients and the lost to follow-up ones at the major baseline characteristics (Supple. Table 1). The mean age at last follow-up was 40.27 ± 23.27 months, with a mean BCVA (log MAR) of 1.07 ± 0.53. The distribution of the included patients with age periods is shown in Fig. 2. The mean period of follow-up was 31.07 ± 19.36 months. One hundred and twenty-eight eyes (72.7%) were operated on in patients less than or equal to 6 months in age. Forty-eight eyes (27.3%) were operated on in patients over 6 months in age. One hundred and sixty-four eyes (93.2%) underwent cataract extraction, and twelve eyes were subjected to cataract surgery with IOL implantation. The BCVA distribution of the included patients at the last follow-up is listed in Table 1, followed by the basic assessed variables in Table 2. Various PCs are described in detail in Table 3. All PCOs were removed using Nd:YAG laser capsulotomy immediately after being detected. Elevated IOP and glaucoma were controlled with drugs.

### Multivariable GEE Analysis

The multivariable GEE analysis indicated that the following factors were positively correlated with BCVA (logMAR): PC (standardized β = 0.076, P < 0.041) and age at primary surgery (standardized β = 0.471, P < 0.001). Conversely, age at last follow-up (standardized β = −0.528, P < 0.001) was negatively correlated with BCVA (logMAR). No significant associations were found between BCVA and gender, surgical procedure, follow-up frequency, or spectacle change frequency. These results are shown in Table 4.

### Partial Correlation Analysis

**Figure 3:**
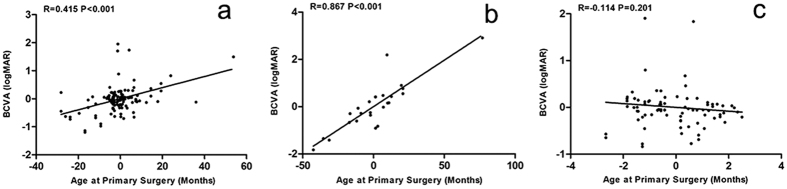
Partial correlation analysis of the relationship between age at primary surgery and BCVA (logMAR). (**a**) Relationship between all ages at primary surgery and BCVA (n = 176). (**b**) Relationship between age at primary surgery (>6 months) and BCVA (n = 48). (**c**) Relationship between age at primary surgery (≤6 months) and BCVA (n = 128). The X-axis represents the residual values of BCVA (logMAR) regressed upon other influencing factors; The Y-axis represents the residual values of age at primary surgery regressed upon other influencing factors. The regression lines were created based on linear regression, and the corresponding amount of variance (R^2^) was accounted for by the linear relationship.

A partial correlation analysis was further performed to determine the linear correlation between surgery age and visual outcome. The results indicated that age at primary surgery was positively correlated with BCVA when the other related factors were considered as a control factor, both for the whole age range (R = 0.415, P < 0.001) (Fig. 3a) and age >6 months (R = 0.867, P < 0.001) (Fig. 3b); however, no significant statistical correlation was noted between age at primary surgery (≤6 months) and BCVA (R = −0.114 P = 0.201) (Fig. 3c).

## Discussion

CC is the most common cause of childhood blindness worldwide^17^,^18^. Although visual acuity can be restored by early surgical intervention in children with CC, visual outcomes after cataract surgery vary widely and are not optimistic. This is the first ever prospective cohort study to evaluate potential related factors that might affect visual outcomes of bilateral CC patients who have undergone primary cataract surgery. The results of this study will be of clinical significance to CC management, involving the surgical timing, approach, and control of complications, to achieve an ideal visual outcome.

This may be the first study reporting visual outcomes of bilateral total congenital cataract patients in China. A previous study by Solebo reported that only 36% (35/97) of children with bilateral cataract achieved a restoration of vision to within the normal range based on age^19^. In a study by Khanna, 17.2% (37/215) of children with bilateral cataracts had visual acuities of less than 0.1, and nearly half of the eyes had visual acuities of greater than 0.3.15 In our series, 52.8% (93/176) of the eyes had a BCVA of less than 0.1, while only 10.8% (19/176) of the eyes had a BCVA of greater than 0.3 after bilateral cataract extraction^20^. The poor visual outcome ratio of 93/176 (52.8%) ≤ 0.1 in this study was worse than the results from South India (17.2% ≤ 6/60)^20^, Maharashtra, India (48.6% ≤ 6/60)^15^. Otherwise, the relatively favorable visual outcome ratio of 19/176 (10.8%) ≥ 0.3 was smaller than results from South Africa (24.7% ≥ 6/18)^16^, Bangladeshi (33% ≥ 6/18)^14^, Tanzanian (58% ≥ 6/18)^12^, UK (40.6 ≥ 6/18)^13^, Mexico (40% ≥ 6/18)^21^, Nepal (36.6% ≥ 6/18)^22^. The large discrepancy in visual outcome between our study and others may be due to differences in follow-up duration, cataract morphology, physical age and any other complicated related factors.

Treatment for CC is long-term, complex, and intensive. Complicated factors may affect the visual outcomes following CC surgery^20^. The results of multivariable GEE analysis demonstrated that PCs, age at last follow-up and age at primary surgery were significantly associated with BCVA (logMAR). PC was indicated as an important related factor of BCVA (logMAR) in our study. Indeed, the management of PCs is challenging for pediatric ophthalmologists due to their variety and high occurrence rate. Postoperative inflammatory responses in children can lead to fibrinous reactions, pigment deposits on IOLs, IOL decentration and posterior synechiae. Even capsular blockage syndrome has been reported after pediatric cataract surgery^23^. Secondary glaucoma, a feared complication of pediatric cataract surgery, is commonly observed in infants. It is also notable that the rate of progression from ocular hypertension to aphakic glaucoma was determined to be 23% over a mean observation period of 7.2 years^24^. Moreover, numerous studies have reported PCO rates ranging from 50% to nearly 100%^4^,^5^,^25^. In a study by Whitman, the incidence of retinal detachment was found to be 3.2% (33/1017) over a mean follow-up of 6.8 years (range, 2 to 18.3 years)^26^. In the present study, there was a 0.6% incidence of secondary glaucoma, an 11.9% incidence of ocular hypertension (OH) and a 22.2% incidence of PCO. There were no cases of fibrinous membrane or RD. Although progressive technique and proper management have been proven to be effective in preventing or delaying the occurrence of complications, unavoidable PCs remain a major obstacle to achieving good visual outcomes following CC surgery^9^.

Age at last follow-up has long been considered an important physiological factor that affects the visual outcomes of CC patients. The common understanding of the most critical period for visual development is within the first 6 weeks of life, during which, vision is subcortically mediated and infants are relatively resistant to amblyopia^27^. However, recent studies claim that the sensitive period of visual development can continue to progress until 12 years of age^28^. In our study, the highest correlation coefficient (|standardized β| = 0.528, P < 0.001) was found between age at last follow-up and BCVA (logMAR), indicating the powerful plasticity in visual development in the enrolled CC patients. This result suggests meaningful monitoring and therapeutic value for these patients during their first decade of life.

The second highest correlation coefficient (**|**standardized β**|** = 0.417, P < 0.001) between age at primary surgery and BCVA (logMAR) demonstrated that age at primary surgery was a crucial factor that may influence visual outcomes. Age at surgery was a relatively controllable related factor for the ophthalmologist and provided significant clinical guidance for surgical intervention; therefore, a partial correlation analysis between age at surgery and visual outcome was performed. The results demonstrated that age at primary surgery was positively correlated with BCVA (logMAR); as such, a younger age at primary surgery is related to better postoperative visual acuity. Similar results were reported by Hartman, who evaluated age at primary surgery as a singular variable^29^. The linear trend between age at primary surgery and BCVA confirmed the importance of early surgical intervention.

However, surgery at a young age may predispose infantile eyes to more PCs. It has been reported that the complication rate after CC surgery is high, particularly when the surgery is performed early in life^30^,^31^. The linear trend between age at primary surgery and BCVA indicated that cataract surgery must be scheduled earlier to optimize visual outcomes; however, earlier surgeries are associated with a greater prevalence of PCs. Therefore, it is urgent to define the optimal timing for surgery to achieve a favorable risk-benefit ratio. Hussain noted that the visual outcome is significantly better when surgery is performed at the age of 2 to 3 months compared with 3 to 12 months^32^. However, Maurer and Lewis reported only a small correlation between age at surgery and visual outcome in children treated by 5 months of age. At present, the ideal timing of surgery for bilateral CC remains debated, and little is known about the critical period for bilateral deprivation amblyopia in humans^33^. In our study, the majority of patients (72.7%) were operated on during their first 6 months of life; therefore, a separate correlation analysis was performed when the age at primary surgery was less than or equal to 6 months. It was surprising that no significant statistical correlation was found between age at primary surgery (≤6 months) and BCVA (logMAR) when all influencing factors were considered as a control factor. Although the critical period for visual development occurs during the first 6 weeks of life, we found no significant differences in visual outcome when the age at primary surgery was within the first 6 months of life. This result implies a longer critical period for bilateral CC.

The success of cataract surgery in children depends heavily on postoperative care and good compliance with follow-up visits. It is well understood among pediatric ophthalmologists that rigorous and regular follow-up is essential for the successful management of childhood cataract. Follow-up is one of the most important indicators of postoperative compliance. Although our study showed that the follow-up frequency did not significantly affect BCVA (logMAR) (standardized β = −0.144, P = 0.234), a trend of better visual outcome with higher follow-up frequency was identified.

Gender, surgical procedure, and spectacle change frequency did not significantly affect BCVA (logMAR) in our series. It has previously been reported that no significant associations exist between BCVA and gender in children undergoing cataract surgery, which was similar to our results. The timing of IOL implantation is still controversial at present. Kim noted that early cataract surgery, aphakic correction with glasses and secondary IOL implantation at approximately 2 years of age is an appropriate method of managing CC^34^. Birch indicated that IOLs and aphakic contact lenses support the development of similar levels of visual acuity following unilateral cataract surgery^35^. Our results suggested that there were no significant associations between surgical procedure (cataract surgery with/without IOL implantation) and visual outcome, which is similar to Birch’s research. In our series, only 12 eyes/6 patients underwent cataract extraction and IOL implantation at a later age (age at primary surgery: 42.66 ± 16.28 months); these delays were caused by poor financial situations. The association between BCVA and surgical procedure should be further analyzed using a larger sample size in the future. Changes in spectacle prescription are indicative of refractive variation; however, refractive variation does not necessarily affect the visual outcome, which might mostly explain why spectacle change frequency did not significantly affect BCVA (logMAR).

The limitations of our study were the wide age range at follow-up and the relatively small sample size, especially in the number of children undergoing cataract extraction with IOL implantation. Further investigations may be necessary to determine additional factors that influence visual outcome. In conclusion, the results of our study revealed that visual outcome is significantly associated with PCs, age at last follow-up, and age at primary surgery. Better visual outcomes were achieved in children undergoing surgical intervention at an earlier age and with a reduced occurrence of PCs. The results also revealed that age at last follow-up and age at primary surgery were the most crucial factors affecting the visual outcome. In summary, the treatment of CC remains challenging.

## Methods

### Study Design and Patient Recruitment

The present study was designed as a hospital-based, prospective cohort study. Before May 2014, participants were recruited from Zhongshan Ophthalmic Center (ZOC), one of the largest eye hospitals in China, located in Guangzhou City in the southern part of the country. The participants were considered eligible if they were diagnosed with bilateral total CC under a slit-lamp eye examination by an ophthalmologist at ZOC and if they underwent primary cataract surgery before the age of ten years. The exclusion criteria were ocular trauma, infection, congenital glaucoma, anterior segment dysgenesis, optic nerve or other fundus abnormalities, prematurity and cataract associated with other syndromes, and systemic disorders. Patients with less than 1 year of follow-up were also excluded from the study. The study was included in the Childhood Cataract Program of the Chinese Ministry of Health (CCPMOH)^36^,^37^ and was approved by the ZOC Institutional Review Board at Sun Yat-sen University (IRB-ZOC-SYSU), Guangzhou, China. Ethical approval for this study was provided by the ZOC Ethics Committee at Sun Yat-sen University and followed the tenets of the Declaration of Helsinki. Informed consent was collected from either the patients’ guardians or the patients themselves.

### Surgical Technique

Pre-operative examinations, including visual acuity, fundus examination, retinoscopy, keratometry and B-scan ultrasonography, were performed. The subjects’ parents received detailed explanations of the preoperative workup, surgery, anesthesia, and all components of the postoperative management. Cataract surgery with/without IOL implantation was performed by two equivalently experienced pediatric ophthalmologists (YZL and WRC). Variations in surgical technique occurred as a function of patient age, timing of the surgery and degree of posterior capsule opacity. Standard scleral tunnel incision was employed. An anterior capsulotomy was made in a continuous curvilinear fashion. The nucleus and cortex were removed using either a manual irrigation/aspiration device or an automated vitrectomy instrument. The vitrectomy instrument was used to create a central posterior capsulotomy and perform a limited anterior vitrectomy in selected cases. All of the surgeries were performed under general anesthesia.

### Postoperative Regimen

The standard postoperative regimen consisted of 2 mg of subconjunctival dexamethasone and Tobradex eye drops (tobramycin 0.3%, dexamethasone 0.1%; Alcon, USA) 4 times per day and Tobradex eye ointment (tobramycin 0.3%, dexamethasone 0.1%; Alcon, USA) once per night for 1 month. Subsequently, anti-inflammatory steroid drugs were replaced with steroid-free pranoprofen eye drops 4 times per day (pranoprofen 0.1%; Senju Pharmaceutical Co., Ltd., Japan) for another month.

### Follow-up Protocol

The follow-up protocol after surgery was consistent with our previous study^37^. The protocol called for visits at 1 week, 1 month, 2 months and 3 months after surgery and then every 3 months. At each postoperative visit, BCVA assessment and/or retinoscopy were performed, as well as a slit lamp examination and a fundus examination, if possible. Study outcomes were assessed annually and included gender, presenting age, age at primary surgery, BCVA, PC, follow-up frequency and spectacle change frequency. BCVA was evaluated using the Teller Acuity Card Test (Vistech, Dayton, Ohio, USA) in preverbal children or the Snellen chart or “E” charts in older children. The results were converted to the logarithm of the minimum angle of resolution (LogMAR) for statistical analysis^3^,^38^. The BCVA was recorded at the last follow-up visit after surgery. PCs, such as fibrinous membrane, ocular hypertension (OH), glaucoma, retinal detachment, and posterior capsule opacification (PCO), were recorded. The timing of follow-up visits was calculated from the daily lists of appointments maintained in the clinic, and the timing of changes in spectacle prescription was collected from the optometry center in the hospital. From these data, the follow-up frequency and spectacle change frequency per year were calculated.

### Statistical Analysis

Data were analyzed using the computer package SPSS (Version 19.0; SPSS, Inc., Chicago, IL). Bilateral eyes from the same patient were included for analysis. Therefore, we employed multivariable generalized estimating equations (GEEs) to estimate the effects of different factors on BCVA (logMAR) using a working covariance matrix of unstructured correlations for nested data. Partial correlation is mathematically equivalent to the correlation between the residual scores of the two variables regressed upon the control variable^39^. A partial correlation analysis was performed to evaluate the relationship between BCVA (logMAR) and age at primary surgery, using the other related factors as a control. A P value less than 0.05 was considered significant.

## Additional Information

**How to cite this article**: Zhang, L. *et al*. Visual Outcome and Related Factors in Bilateral Total Congenital Cataract Patients: A Prospective Cohort Study. *Sci. Rep*. **6**, 31307; doi: 10.1038/srep31307 (2016).

## Supplementary Material

Supplementary Information

## Figures and Tables

**Table 1:** BCVA distribution of the included patients at last follow-up.

BCVA at last follow-up	Number of eyes (%)
BCVA < 0.1	93 (52.8)
0.1 ≤ BCVA < 0.3	64 (36.4)
0.3 ≤ BCVA < 0.7	13 (7.4)
0.7 ≤ BCVA	6 (3.4)
Total	176 (100)

BCVA = Best corrected visual acuity.

**Table 2:** Factors potentially related to BCVA in the included patients.

Factors	Numbers (%)
Gender (n = 88)	
Male	58 (65.9)
Female	30 (34.1)
Surgical procedure (n = 176)	
CE	164 (93.2)
CE+IOL	12 (6.8)
Postoperative complications (n = 176)	52 (29.5)
	Mean ± SD (range)
Age at last follow-up (months)	40.27 ± 23.27 (15–138)
Age at primary surgery (months)	8.79 ± 13.59 (2–100)
Follow-up frequency (times)	4.57 ± 1.96 (1.32–9.93)
Spectacle change frequency (times)	1.57 ± 1.09 (0.25–6.00)

CE = Cataract extraction; CE + IOL = Cataract extraction and IOL implantation.

**Table 3:** Postoperative complications.

Complications	No. of Eyes (n = 176) (%)
Ocular hypertension*	21 (11.9)
Fibrinous membrane	0 (0)
Secondary glaucoma	1 (0.6)
Retinal detachment	0 (0)
Posterior capsule opacification*	39 (22.2)
Total	52 (29.5)

^*^There were nine eyes with both ocular hypertension and posterior capsule opacification, accounting for the total number of 52.

**Table 4:** Multivariable GEE analysis of the factors influencing the BCVA (logMAR) after surgery (n = 176).

Influencing factors	Standardized β	Wald *χ*^2^	P
Gender	0.106	0.229	0.633
Age at last follow-up	−0.528	29.334	<0.001*
Age at primary surgery	0.417	20.428	<0.001*
Surgical procedure	−0.437	1.089	0.297
Postoperative complications	0.076	4.173	0.041*
Follow-up frequency	−0.144	1.419	0.234
Spectacle change frequency	−0.069	0.819	0.366

*P < 0.05, indicating significant effects of the factors on BCVA (logMAR).
