# Supplementary material for: Visual Outcome and Related Factors in Bilateral Total Congenital Cataract Patients: A Prospective Cohort Study
Source: Sci Rep. 2016 Aug 3;6:31307. doi: 10.1038/srep31307 (PMC4971510; doi:10.1038/srep31307)
Supplement: Supplementary Information [file srep31307-s1.pdf]

Supplementary information

Title Page

Visual Outcome and Related Factors in Bilateral Total Congenital Cataract Patients: A Prospective Cohort Study

Li Zhang+, Xiaohang Wu+, Duoru Lin, Erping Long, Zhenzhen Liu, Qianzhong Cao, Jingjing Chen, Xiaoyan Li, Zhuoling Lin, Lixia Luo, Hui Chen, Wu Xiang, Jinchao Liu, Xuhua Tan, Bo Qu, Haotian Lin*, Weirong Chen*, Yizhi Liu

[+]These authors contributed equally to this work

Institution: State Key Laboratory of Ophthalmology, Zhongshan Ophthalmic Center, Sun Yat-sen University, Guangzhou, Guangdong, 510060, People's Republic of China

[§]**Co-corresponding authors**: Haotian Lin, M.D., Ph.D., Email: gddlht@aliyun.com; Weirong Chen, M.D., E-mail: chenwr_q@aliyun.com; Address: Zhongshan Ophthalmic Center, Xian Lie South Road 54#, Guangzhou, China, 510060. Telephone number: +86-020-87330493, Fax: +86-020-87333271.

Supple. Table 1. Comparison of the included patients vs. the lost to follow-up ones at the major baseline characteristics

Baseline	Included patients (88 person ,176 eyes)	Lost to follow-up patients (39 person,78 eyes)	Statistics	P value
**Gender** (Male/Female)	58/30 (n=88)	27/12 (n=39)	x^2=0.135*	0.714
**Surgical procedure** CE/(CE＋IOL)	164/12 (n=176)	68/10 (n=78)	x^2=2.461*	0.117
**Age at primary surgery** (Months, mean± SD)	8.79±13.59 (n=88)	13.48±16.94 (n=39)	t=-1.766$^{\$}$	0.080

CE = Cataract extraction; CE + IOL = Cataract extraction and IOL implantation.

*Chi-squared test was used; $^{\$}$T test was used.

p<0.05 indicates statistically significant different.
